# The Hepatic Mitochondrial Alterations Exacerbate Meta-Inflammation in Autism Spectrum Disorders

**DOI:** 10.3390/antiox11101990

**Published:** 2022-10-07

**Authors:** Giovanna Trinchese, Fabiano Cimmino, Gina Cavaliere, Angela Catapano, Chiara Fogliano, Adriano Lama, Claudio Pirozzi, Claudia Cristiano, Roberto Russo, Lidia Petrella, Rosaria Meli, Giuseppina Mattace Raso, Marianna Crispino, Bice Avallone, Maria Pina Mollica

**Affiliations:** 1 Department of Biology, University of Naples Federico II, 80126 Naples, Italy; 2 Department of Pharmaceutical Sciences, University of Perugia, 06123 Perugia, Italy; 3 Department of Pharmacy, University of Naples Federico II, 80131 Naples, Italy; 4 Task Force on Microbiome Studies, University of Naples Federico II, 80138 Naples, Italy

**Keywords:** autism spectrum disorders, inflammation, liver, mitochondrial dysfunction

## Abstract

The role of the liver in autism spectrum disorders (ASD), developmental disabilities characterized by impairments in social interactions and repetitive behavioral patterns, has been poorly investigated. In ASD, it has been shown a dysregulation of gut–brain crosstalk, a communication system able to influence metabolic homeostasis, as well as brain development, mood and cognitive functions. The liver, with its key role in inflammatory and metabolic states, represents the crucial metabolic organ in this crosstalk. Indeed, through the portal vein, the liver receives not only nutrients but also numerous factors derived from the gut and visceral adipose tissue, which modulate metabolism and hepatic mitochondrial functions. Here, we investigated, in an animal model of ASD (BTBR mice), the involvement of hepatic mitochondria in the regulation of inflammatory state and liver damage. We observed increased inflammation and oxidative stress linked to hepatic mitochondrial dysfunction, steatotic hepatocytes, and marked mitochondrial fission in BTBR mice. Our preliminary study provides a better understanding of the pathophysiology of ASD and could open the way to identifying hepatic mitochondria as targets for innovative therapeutic strategies for the disease.

## 1. Introduction

The prevalence of autism spectrum disorders (ASD) has dramatically increased in the last decade. ASD is a developmental disability characterized by persistent impairments in social interaction and restricted, repetitive patterns of behaviors, interests, and activities [1]. The etiopathogenesis of ASD is multifactorial including genetic, environmental and immunological aspects [2], combined with neurotrophic dysregulation and increased sensitivity to oxidative stress [3]. The core features of ASD are associated with immune dysfunctions both at the systemic and cellular level [4]. 

In individuals with ASD, several lines of evidence point to ongoing inflammation in the brain [5] as well as in the peripheral tissues [4,6]. Indeed, increased pro-inflammatory cytokines including IL-1β, IL-6, and TNFα were found in both sera [6,7] and post-mortem brains of ASD patients [8]. A low-grade chronic inflammatory state and oxidative stress are considered causal factors for noncommunicable diseases, including obesity and neurodegenerative diseases [9,10]. Interestingly, findings suggest that ASD contributes to the risk of obesity [11]. Thus, the altered metabolic and inflammatory pathways may be targeted to develop a potential innovative treatment for ASD [9].

Several studies highlighted the role of the gut–brain axis, a complex communication system between microbiota and the central nervous system (CNS), not only in maintaining metabolic and immune homeostasis but also in influencing brain development, mood and cognitive functions [12]. Inflammatory gastrointestinal diseases are often reported as a comorbidity in ASD [3]. Accordingly, in an animal model of ASD (BTBR mice), researchers observed a marked intestinal dysbiosis, increased levels of pro-inflammatory factors and leaky gut associated with impaired behavior, neuroinflammation and immune system dysregulation [13,14]. In this crosstalk between gut and CNS, the liver represents the crucial metabolic organ performing biochemical functions necessary for whole-body health, energy balance and metabolic state [15]. The direct exposure of the liver to increasing amounts of bacteria-derived factors, such as endotoxins, pro-inflammatory cytokines, and lipids, seems to be critical in the systemic and central inflammation [16]. Interestingly, the portal blood delivers not only nutrients to the liver but also numerous products and factors derived from the gut and visceral adipose tissue [17] which are able to modulate the hepatic metabolism. These endocrine and immune mediators, in turn, influence the bioenergetic regulation of hepatic mitochondria.

Indeed, mitochondria play a key role in the regulation of healthy metabolism, supplying the cell with ATP but also producing key molecules involved in metabolism, oxidative stress, and inflammatory processes [9]. Notably, several studies both in human and animal models have shown that mitochondrial dysfunctions are involved in ASD [18,19,20]. Interestingly, mitochondria are very vulnerable to environmental factors and potentially can be considered pivotal mediators of environmental-genetic interactions [21]. It has been recently reported that mitochondrial dysfunctions affect the brain in animal models of autism [22]. Variations in mitochondrial DNA sequence and copy number are associated with ASD [23], and toxins linked to ASD inhibit mitochondrial function [24]. However, it is not fully elucidated whether the atypical functions of mitochondria are the cause or the consequences of ASD. In our work, we analyzed the hepatic mitochondrial metabolism in BTBR T^+^ tf/J (BTBR) mice, which is a validated animal model that displays multiple behavioral phenotypes characterizing ASD, including impaired sociability, communication deficits, and repetitive/stereotypic behaviors [25]. The inbred mouse strain BTBR T^+^ tf/J exhibits a 100% absence of the corpus callosum, a severely reduced hippocampal commissure and a deletion in the ITPR3 gene encoding the type 3 inositol 1,4,5-triphosphate receptor (IP3) [3]. Given that IP3 receptors can mediate interactions between mitochondria and the endoplasmic reticulum [26], this deletion could potentially have an effect on mitochondrial function in BTBR mice [27]. Moreover, ITPRs are intracellular calcium release channels [28,29], and several liver functions are regulated by intracellular calcium. Thus, changes in the expression level of ITPR are found in several hepatic disorders, such as cholestasis and non-alcoholic fatty liver disease [30,31], and they could be linked to mitochondrial dysfunction.

In this study, we highlighted the role of hepatic mitochondria in modulating the crosstalk between inflammatory state and oxidative stress, which are two interdependent processes closely related to metabolic alterations, energy balance and hepatic steatosis [32]. Understanding this interplay can be useful in planning new therapeutic intervention strategies focused at correcting metabolic dysregulation and immune impairment in ASD.

## 2. Materials and Methods

### 2.1. Reagents 

The analytical-grade chemicals used were purchased from Sigma (St. Louis, MO, USA) unless otherwise specified. The standard laboratory chow diet (Mucedola Srl., Milan, Italy) had 60.4% carbohydrates, 29% protein, 10.6% fat, and contained 15.8 kJ/g, determined by bomb calorimeter. 

### 2.2. Animal and Experimental Design

C57Bl/6J (control) and BTBR T^+^ tf/J (BTBR) male mice were purchased from the Jackson Laboratory (Bar Harbor, ME, USA), and colonies were established and maintained in our animal facility. All animals (control, *n* = 7; BTBR, *n* = 7) were housed in a room maintained at 22 °C, on a 12 h:12 h light:dark cycle with ad libitum access to water and standard laboratory chow diet. To minimize litter effects, we used for our experiments male mice 3–4 months of age from at least 3 different mating pairs in each experiment. All experimental procedures were carried out in compliance with the international and national law and policies (EU Directive 2010/63/EU for animal experiments, ARRIVE guidelines and the Basel declaration including the 3R concept) and approved by the Italian Ministry of Health under protocol no.1226/2020-PR. The mice were anaesthetized by an intraperitoneal injection of chloral hydrate (40 mg/100 g body weight) and sacrificed by decapitation. Blood was taken from the inferior cava and portal vein; serum was obtained by centrifuging at 1000× *g* for 10 min and stored at −80 °C for biochemical analyses. The liver was quickly removed, and part of it was immediately used for mitochondrial extraction. The remaining part of the liver was cut into 8 blocks of 1 mm^3^, belonging to different areas fixed for electron microscopy. Other aliquots were frozen and stored at −80 °C for further determinations. 

### 2.3. Body Composition and Energy Balance

During treatments, body weight and food intake were monitored daily to calculate body weight gain and gross energy intake. Body composition assessments were carried out immediately after animals sacrifice as previously reported [33]. The gross energy density for the standard diet (15.88 kJ/g), as well as the energy density of the carcasses, were determined by bomb calorimeter (Parr adiabatic calorimeter, Parr Instrument Co., Moline, IL, USA).

### 2.4. Evaluation of Metabolic Parameters and Inflammatory Markers in Serum and Tissue

Serum concentrations of triglycerides, cholesterol, alanine aminotransferase (ALT) and aspartate transaminase (AST) were measured by the colorimetric enzymatic method using commercial kits (SGM Italia, Rome, Italy and Randox Laboratories Ltd., Crumlin, UK). Commercially available ELISA kits were used to determine serum monocyte chemoattractant protein-1 (MCP-1) (Biovendor R&D, Brno, Czech Republic), serum leptin and adiponectin (B-Bridge International, Mountain View, CA, USA), serum and tissue TNF-α, IL-1β and IL-6 (Biovendor R and D, Brno, Czech Republic (TNF-α); Thermo Scientific, Rockford (IL-1β and IL-6)). Lipopolysaccharide (LPS) was measured using the Limulus amebocyte lysate (LAL QCL-1000; Lonza Group Ltd., Basel, Switzerland) technique. 

### 2.5. Hepatic Histological Analyses

For each animal (*n* = 4) of all analyzed groups, the liver was cut into 8 blocks of 1 mm^3^, belonging to different areas. Each block was fixed and processed according to Cimmino et al. [34]. Semi-thin sections (1.5 μm) were cut with a glass knife for light microscopy observations and stained with 1% toluidine blue solution. PAS staining was performed treating the sections in 1% periodic acid Schiff’s reagent, staining the nuclei with 1% aniline blue, and ultimately washing in 7% acetic acid solution. The sections were observed with a ZEISS Axiocam microscope camera applied to a Zeiss Axioskop microscope. For each animal (*n* = 4/group), images at 20× magnification of 30 serial sections, taken 1 every 10, belonging to each liver blocks, were used to set up the count of Kupffer cells. Ultra-thin sections (50–80 nm) were loaded on 200-mesh grids and observed in a Fei-Tecnai- G2 electron microscope at 120 kV. The measures of mitochondria length were made on images at original magnification 4000× of 30 serial sections, taken 1 every 10, and analyzed using the software Image J 1.45.

### 2.6. Western Blot Analysis

Liver was homogenized at 4 °C in lysis buffer (20 mM Tris–HCl, pH 7.5, 10 mM NaF, 150 mM NaCl, 1% Nonidet P-40, 1 mM phenylmethylsulphonyl fluoride, 1 mM Na_3_VO_4_, leupeptin, and trypsin inhibitor 10 µg/mL). Total protein lysates were obtained as supernatant by centrifugation at 14,000× *g* for 15 min at 4 °C. Protein concentrations were estimated by the Bio-Rad protein assay using free bovine serum albumin (BSA) as standard. An equal amount of protein was subjected to SDS-PAGE and electro transferred onto a nitrocellulose membrane (Amersham Biosciences, Little Chalfont, Buckinghamshire, UK) using a Bio-Rad Transblot (Bio-Rad, Milan, Italy). Membranes were blocked at room temperature in milk buffer (1X PBS, 10% *w/v* non-fat dry milk, 0.1% *v/v* Tween-20) and probed with anti-Binding Immunoglobulin Protein (BIP) (dilution 1:1000), anti-phospho eukaryotic translation initiation factor 2A (p-EIF2α) (dilution 1:1000) and EIF2α (dilution 1:1000), anti-mitofusin1 (MFN1) (dilution 1:500), anti-mitofusin2 (MFN2) (dilution 1:1000), anti-optic atrophy protein (OPA1) (dilution 1:1000), anti-dynamin-related protein (DRP1) (dilution 1:1000), anti-fission 1 protein (FIS1) (dilution 1:1000) (Santa-Cruz Biotechnology, Dallas, TX, USA); anti-phospho-adenosine monophosphate-activated protein kinase-α (p-AMPKα) (dilution 1:1000) and AMPK (dilution 1:1000) (Cell Signaling, Danvers, MA, USA); anti-phospho-acetyl-CoA carboxylase (p-ACC) (dilution 1:1000) and ACC (dilution 1:1000), anti-carnitine-palmitoyl-transferase1 (CPT1) (dilution 1:1000) (Santa-Cruz Biotechnology, Dallas, TX, USA). Western blot using anti-β-actin antibody (dilution 1:1000; Cell Signaling, Danvers, MA, USA) was performed to normalize the protein expression levels. Signals were visualized by chemiluminescence (ECL, Millipore, Burlington, MA, USA) on autoradiographic film (Fujifilm X-ray Film).

### 2.7. Mitochondrial Preparation and Analysis

Liver aliquots were finely minced and washed in a medium containing 100 mM KCl, 50 mM Tris-HCl, pH 7.5, 5 mM MgCl_2_, 1 mM EDTA, 5 mM EGTA, and 0.1% (*w/v*) fatty acid free BSA. Mitochondrial preparation and oxygen consumption analyses, measured polarographically by a Clark-type electrode, were conducted as previously reported [25]. The degree of coupling was determined in mitochondria as previously reported [33] by applying Equation (11) by Cairns et al. [35]: Degree of coupling = √1 − (Jo)sh/(Jo)unc, where (Jo)sh is the oxygen consumption rate in the presence of oligomycin that inhibits ATP synthase, and (Jo)unc is the uncoupled rate of oxygen consumption induced by carbonyl cyanide 4-(trifluoromethoxy)phenylhydrazone (FCCP), which dissipates the trans-mitochondrial proton gradient. (Jo)sh and (Jo)unc were measured as above using succinate (10 mmol/L) + rotenone (3.75 μmol/L) in the presence of oligomycin (2 μg/mL) or FCCP (1 μmol/L), respectively. The carnitine–palmitoyl–transferase (CPT) activity was determined as previously reported [15]. 

### 2.8. Hepatic Oxidative Stress, Antioxidant/Detoxifying Defenses and Lipid Peroxidation

In hepatic mitochondria, we assessed the rate of mitochondrial hydrogen peroxide (H_2_O_2_) release tracking the linear increase in fluorescence (excitation 312, emission 420 nm) caused by the oxidation of homovanillic acid in the presence of horseradish peroxidase [36]. Aconitase activity in hepatic mitochondria was measured in a medium containing 30 mM sodium citrate, 0.6 mM MnCl_2_, 0.2 mM NADP, 50 mM TRIS-HCl pH 7.4, and 2 units of isocitrate dehydrogenase. The formation of NADPH was determined spectrophotometrically (340 nm) at 25 °C. The level of aconitase activity measured equals the basal level of active aconitase. Aconitase inhibited by reactive oxygen species (ROS) in vivo was reactivated by incubating mitochondrial extracts in a medium containing 50 mM dithiothreitol, 0.2 mM Na_2_S, and 0.2 mM ferrous ammonium sulfate; thus, it was possible to determine the total aconitase activity [37]. The specific activity of superoxide dismutase (SOD) was tested in a medium containing 0.1 mM EDTA, 2 mM KCN, 50 mM KH_2_PO_4_, pH 7.8, 20 mM cytochrome c, 5 mM xanthine, and 0.01 U of xanthine oxidase. Measurements were carried out spectrophotometrically (550 nm) at 25 °C by monitoring the decline in the reduction rate of cytochrome c by superoxide radicals. One unit of SOD activity is defined as the concentration of enzyme that inhibits cytochrome c reduction by 50% in the presence of xanthine + xanthine oxidase system [38]. We measured the following in liver homogenate: reduced (GSH) and oxidized (GSSG) glutathione concentrations, the levels of malondialdehyde (MDA) as an indicator of lipid peroxidation, and the protein carbonyl accumulation (PC) as an indicator of protein oxidative modification as previously indicated [34]. The enzymatic activities of glutathione S-transferases (GST) and NAD(P)H-quinone-oxidoreductase (NQO1) were evaluated spectrophotometrically in cytoplasmic extracts [39]. 

### 2.9. Statistical Analysis

All values were reported as means ± SEM. The unpaired t-test was used to compare the differences among the groups. The differences were considered statistically significant at *p* < 0.05. All analyses were executed with GraphPad Prism 8 (GraphPad Software, San Diego, CA, USA).

## 3. Results

### 3.1. Metabolic and Inflammatory Profile in BTBR Mice

**Figure 1:**
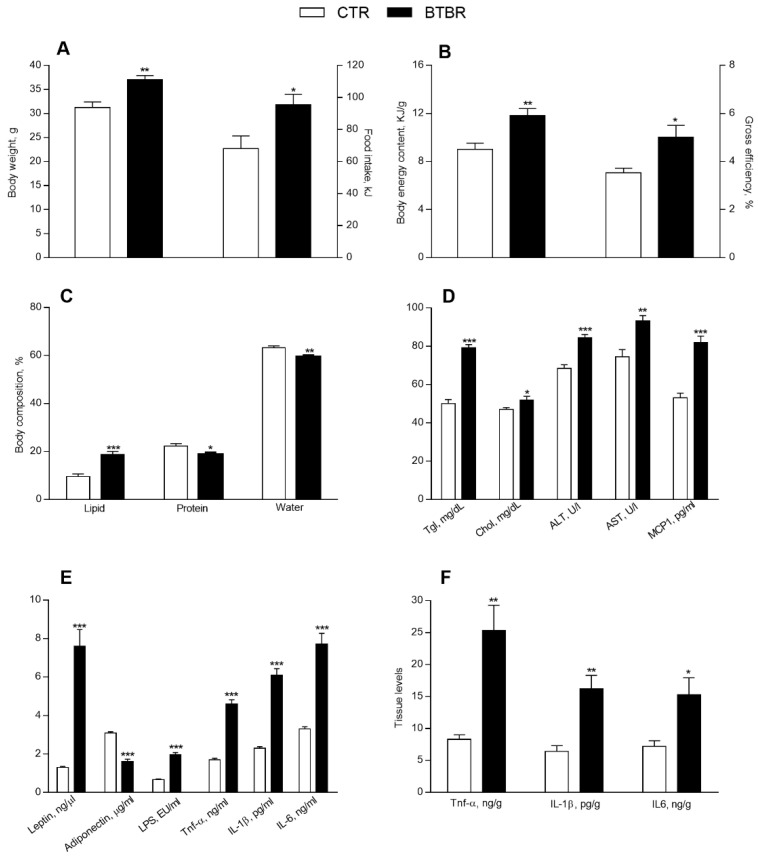
Metabolic and inflammatory profile in control and BTBR mice. Body weight and daily food intake at the end of treatment (**A**). Body energy content and gross efficiency (**B**), body lipid, protein, and water percentages (**C**). Serum levels of triglycerides, cholesterol, alanine aminotransferase (ALT), aspartate aminotransferase (AST), monocyte chemoattractant protein-1 (MCP-1) (**D**), leptin, adiponectin, lipopolysaccharide (LPS), tumor necrosis factor (TNF-α), interleukin-1β (IL-1β), interleukin-6 (IL-6) (**E**). Hepatic levels of TNF-α, IL-1β, IL-6 (**F**). Data are presented as means ± SEM from *n* = 7 animals/group. * *p* < 0.05, ** *p* < 0.01, *** *p* < 0.001 BTBR vs. control (CTR).

BTBR mice, compared to controls, showed a significant increase in body weight, food intake, energy content and gross efficiency (Figure 1A,B). In addition, BTBR mice, compared to controls, displayed a significant increase in lipids percentage (Figure 1C) and a reduction in water and protein percentage (Figure 1C). Serum levels of triglycerides and cholesterol, as well as ALT and AST, markers of hepatic functionality, and MCP-1 were significantly increased in the BTBR mice compared to controls (Figure 1D). BTBR mice, compared to controls, exhibited decreased serum levels of adiponectin and increased serum levels of leptin as well as higher serum levels of systemic inflammatory markers, such as TNF-α, IL-1β, IL-6 and LPS (Figure 1E). The levels of TNF-α, IL-1β and IL-6 were significantly higher also in the liver of BTBR mice compared to controls (Figure 1F), indicating that the BTBR genotype affected the systemic and hepatic inflammation.

### 3.2. Hepatic Morphological Features in BTBR Mice

**Figure 2:**
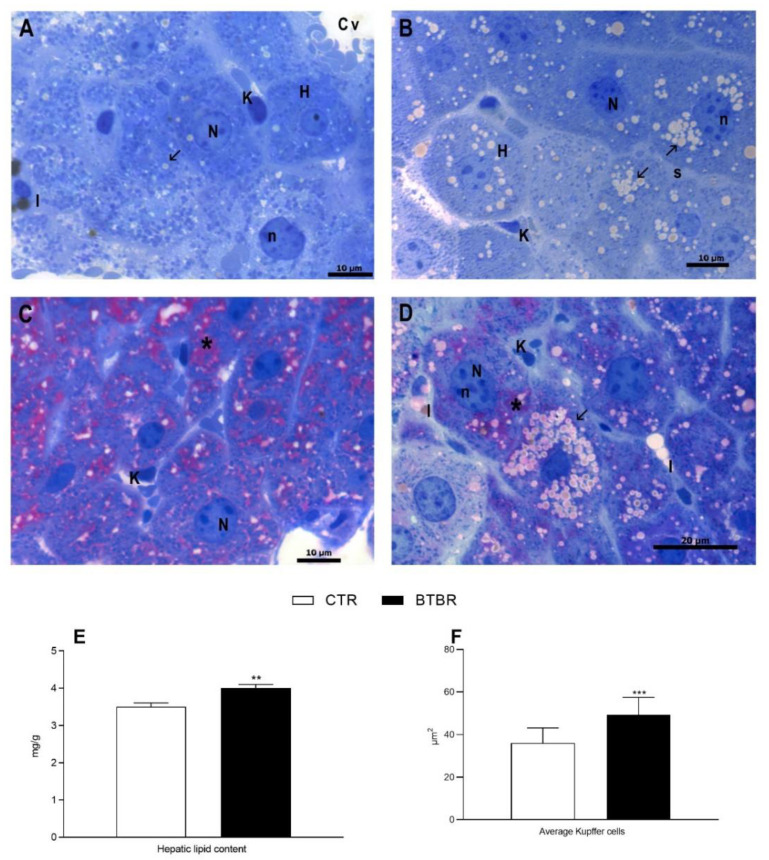
Hepatocytes morphological features of control and BTBR mice. Control group (CTR) (**A**,**C**); BTBR group (**B**,**D**). Toluidine blue staining (**A**,**B**). PAS staining on resin sections (**C**,**D**). CTR shows hepatocytes (H) arranged around the centrilobular vein (Cv) (**A**). BTBR group shows an increase in lipid drops (arrow), irregularly shaped nuclei (N), nucleolar hypertrophy (n) (**B**). PAS staining of CTR revealed widespread positivity (asterisk) (**C**). BTBR hepatocytes show different grade of positivity (asterisk). K: Kupffer cell, I: Ito cell (**D**).(**A**–**D**): 100× magnification. Hepatic lipid content (**E**), and average Kupffer cells (**F**). ** *p* < 0.01, *** *p* < 0.001 BTBR vs. Control (CTR).

The control group shows a normal liver morphology with well-delineated polyhedral hepatocytes radiating from the centrilobular vein in hepatic cords, divided by blood sinusoids in which Kupffer and Ito cells are visible (Figure 2A,C). Hepatocytes display central nucleus with a regular shape and cytoplasm with few lipid droplets (Figure 2A,C). In BTBR mice livers, even though the hepatic architecture is preserved, hepatocytes show micro and macro vesicular steatotic characteristics (Figure 2B,D). Furthermore, numerous hepatic cells exhibit crenulated nuclei, irregularly shaped, as well as the presence of many nucleoli, when compared to control group (Figure 2B,D). In addition, in BTBR mice, we detected a significant increase in total hepatic lipid content and in Kupffer cells (Figure 2E,F). PAS reaction in the control groups shows a widespread localization of glycogen in the cytoplasm of hepatocytes (Figure 2C). In BTBR, the PAS positivity does not seem to have a uniform distribution (Figure 2D).

**Figure 3:**
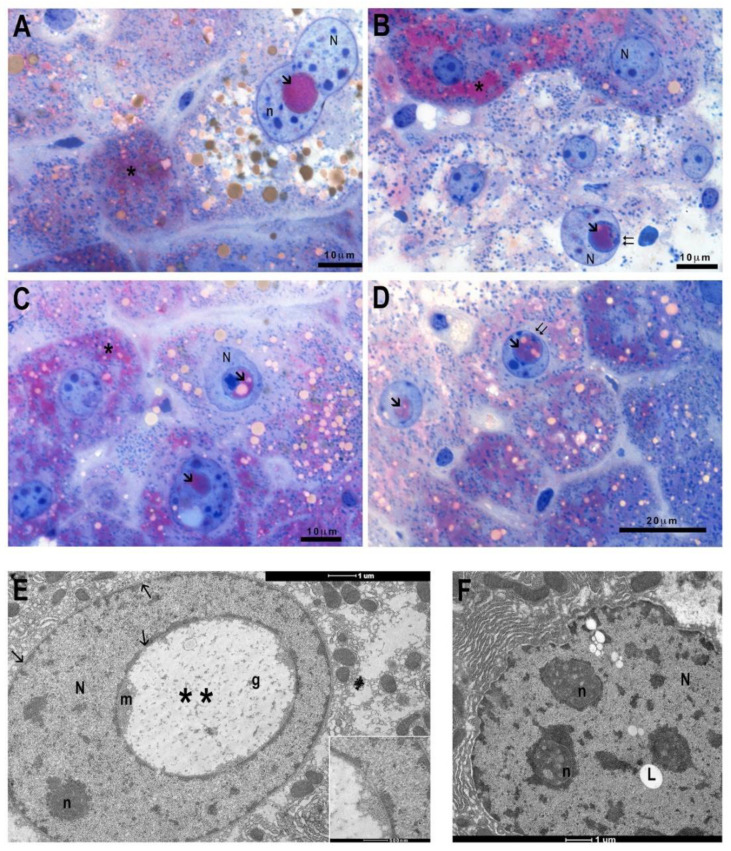
Morphological features of control and BTBR mice liver. PAS staining of BTBR mice liver (**A**–**D**). Some hepatocytes show nuclear inclusions (arrow), with PAS-positive cytoplasm and lipid drops, which at some point maintains contact with the nuclear envelope (double arrow). Transmission Electron Microscopy micrographs of BTBR mice liver (**E**,**F**) highlighting nuclear inclusions (double asterisks) with glycogen (g) or lipid droplets (L). N: nucleus; n: nucleolus; asterisk: PAS positivity. (**A**–**D**): 100× magnification; (**E**,**F**): 7000× magnification.

Furthermore, in BTBR liver sections, it is usual to find nuclei with anomalous nuclear inclusions (NIs), delimited by a membrane, which at some point maintains contact with the nuclear envelope, containing positive PAS-staining cytoplasm and lipid drops (Figure 3A–D). TEM investigations have confirmed, in BTBR sections, the presence of NIs, which appear delimited by nuclear envelope, mainly containing cytoplasm, glycogen, and some organelle debris. It is also possible to find intranuclear lipid drops not delimited by the nuclear envelope (Figure 3E,F).

**Figure 4:**
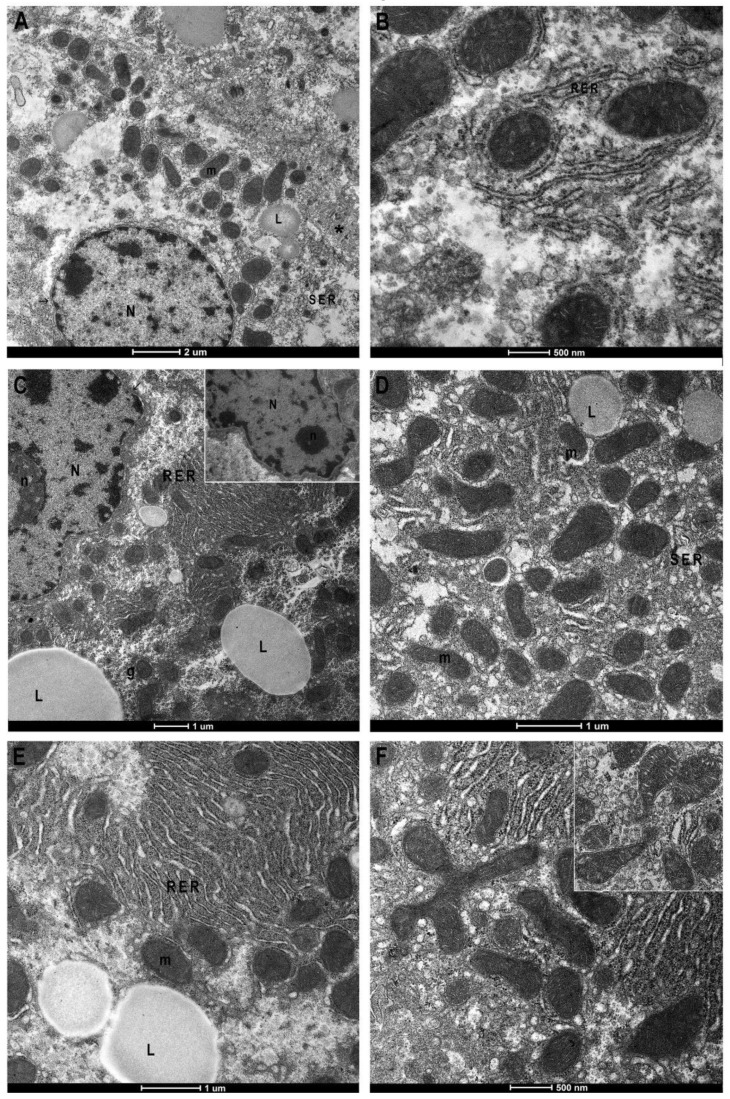
Transmission Electron Microscopy micrographs of control and BTBR mice liver. Control (CTR) (**A**,**B**) and BTBR mice liver (**C**–**F**). CTR liver shows regular nucleus (N), few lipids drop (L), well-preserved mitochondria (m) associated with rough (RER) or smooth reticulum (SER) (**A**). Greater magnification of mitochondria, surrounded by RER (**B**). BTBR liver shows irregular nuclear envelope, hypertrophic nucleoli and RER with congested cisterns, increased number and size of lipid drops (**C**). Mitochondria, mostly small in size (**C**–**E**) and mitochondrial fission phenomena ((**F**) and insert) are recognizable. Asterisk: bile canaliculus; (g) glycogen; (arrow) nuclear pore. Magnification: (**A**) 2550×, (**B**) 9900× (**C**) 4000×, (**D**) 7800×, (**E**) 7000×, (**F**) 9900×.

**Figure 5:**
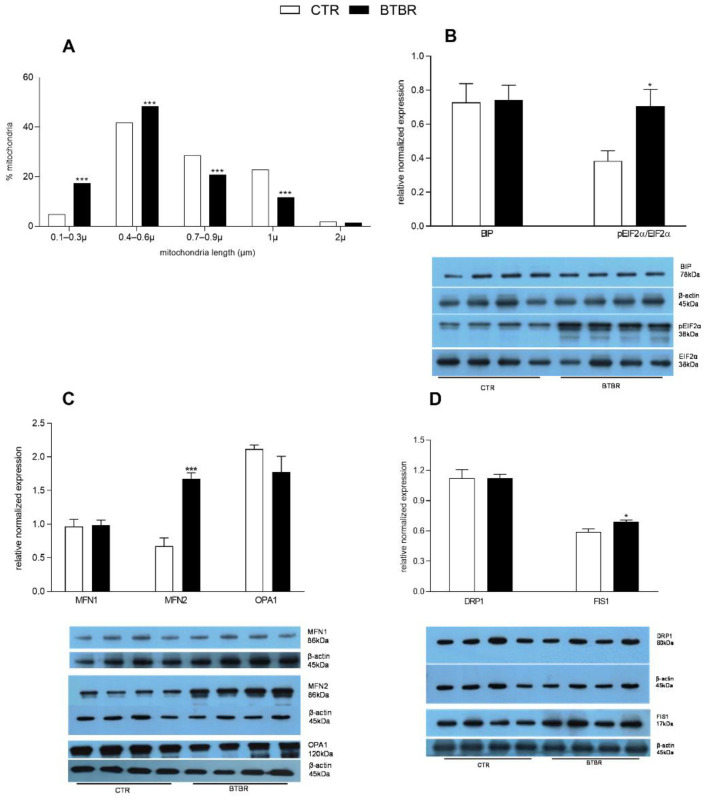
Hepatic markers of the endoplasmic reticulum stress and mitochondrial dynamics in control and BTBR mice. Percentage and length of mitochondria detected by histological analyses (**A**). Hepatic expression levels of endoplasmic reticulum stress markers: Binding Immunoglobulin Protein (BIP) and phosphorylated eukaryotic translation initiation factor 2A (pEIF2α), as ratio pEIF2α/EIF2α (**B**). Expression levels of markers of mitochondrial dynamics: fusion-related proteins mitofusin 1 (MFN1), mitofusin 2 (MFN2) and optic atrophy protein (OPA1) (**C**), and fission-related dynamin-related protein (DRP1) and fission 1 protein (FIS1) (**D**). (*n* = 4 animal/group). * *p* < 0.05, *** *p* < 0.001, BTBR vs. Control (CTR).

The hepatic sections of the control group display, by TEM, the ultrastructural normal characteristics with mono or binucleated cells, well-delineated smooth reticulum, and rare lipid drops, with the presence of microvillar protrusions toward the bile canaliculus (Figure 4A,B). Furthermore, the nucleus has a regular outline with peripheral heterochromatin and abundant euchromatin as well as evident nuclear pores (arrow). The nucleus is surrounded by rough endoplasmic reticulum (ER), and tubular vesicles of smooth ER are observable. Mitochondria with well-defined crests appear surrounded by cisterns of rough ER (Figure 4A,B). Investigations in the BTBR liver highlighted numerous cytological alterations; it confirmed the presence of numerous hepatocytes with crenulated nuclei with strongly irregular outlines, hypertrophied nucleoli (Figure 4C) and an increase in lipid droplets (Figure 4C–E). Moreover, there are also dilated smooth ER profiles (Figure 4F) and hypertrophic rough ER with dilated cisterns at the extremity of which numerous and small mitochondria are associated (Figure 4C–F). Mitochondria preserve a normal morphology, but they are mostly small in size (Figure 4D–F). Mitochondrial fission phenomena are also evident in the histological section (Figure 4F) and confirmed by statistical analysis, where an increased number of small-sized mitochondria was detected in BTBR livers (Figure 5A).

### 3.3. Hepatic Markers of ER Stress and Mitochondrial Dynamics in BTBR Mice

The increased fission in BTBR mice was confirmed by the reduced mitochondrial length (reduced percentage of mitochondria ranging in size from 0.7 to 2 μm) observed by histological analysis (Figure 5A). In the liver of BTBR mice compared with the controls, we detected a higher pEIF2α/EIF2α ratio, while no difference was observed in BIP relative levels (Figure 5B). The expression levels of marker proteins for mitochondrial fusion, MFN1 and OPA, was not significantly different in BTBR compared to the control group, while MFN2 levels significantly increased in BTBR (Figure 5C). Increased mitochondrial fission in the liver of BTBR mice was indicated by higher expression levels of FIS1 compared to controls, although no significant difference was observed in DRP1 expression levels among the two groups (Figure 5D). 

### 3.4. Hepatic Mitochondrial Oxidative Capacities in BTBR Mice

**Figure 6:**
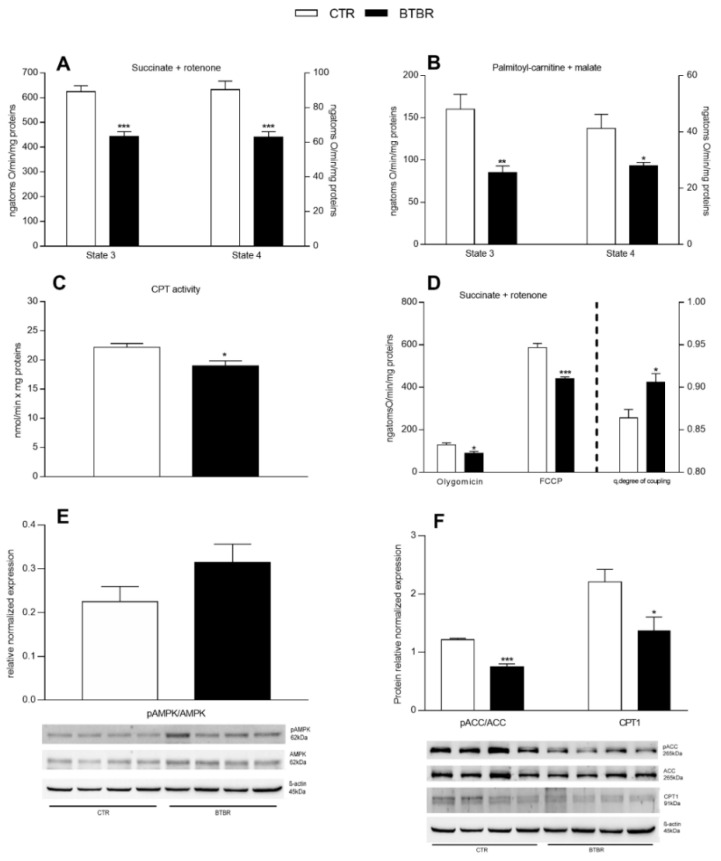
Hepatic mitochondrial function and efficiency, AMPK/ACC and CPT1 expression in control and BTBR mice. Hepatic mitochondrial respiration rates were evaluated with succinate + rotenone (**A**) or palmitoyl-carnitine + malate (**B**) as substrates. CPT activity was measured in isolated mitochondria (**C**). Mitochondrial oxygen consumption in the presence of oligomycin, uncoupled by carbonyl-cyanide-4(trifluoromethoxy)phenylhydrazone (FCCP) and the degree of coupling (**D**) were shown. Hepatic expression levels of phosphorylated adenosine-monophosphate-activated protein kinase-α (pAMPK), as ratio pAMPK/AMPK (**E**), phosphorylated acetyl-CoA-carboxylase (pACC) as ratio pACC/ACC, and carnitine-palmitoyl-transferase 1 (CPT-1) (**F**). *n* = 4. Data are presented as means ± SEM from *n* = 7 animals/group. * *p* < 0.05, ** *p* < 0.01, *** *p* < 0.001, BTBR vs. Control (CTR).

Mitochondria isolated from the liver of BTBR and control mice showed a significant reduction in respiratory parameters (state 4 and state 3) using succinate + rotenone and palmitoyl carnitine + malate as substrates (Figure 6A,B). These results indicated the lower ability of mitochondria from BTBR mice to oxidize both lipid and non-lipid substrates. Moreover, hepatic mitochondria from BTBR mice showed a reduced CPT activity compared to the controls (Figure 6C), indicating a lower transport of fatty acids into the mitochondria. To test mitochondrial efficiency, we measured oxygen consumption in the presence of oligomycin and carbonyl cyanide-p-trifluoromethoxyphenylhydrazone (FCCP). Oligomycin state 4 respiration and maximal FCCP-stimulated respiration showed a significant reduction in BTBR animals compared to the controls (Figure 6D). Hepatic mitochondrial energy efficiency, assessed as the degree of coupling, significantly increased in BTBR mice compared to the controls (Figure 6D, right Y axis). We also investigated the hepatic expression of AMPK and pAMPK since their ratio (pAMPK /AMPK) is indicating the activation level of the enzyme. AMPK is a cellular energy sensor involved in: (i) the control of mitochondrial biogenesis and homeostasis, including the regulation of the AMPK–ACC–malonyl–CoA axis on the control of fatty acid oxidation; (ii) regulation of the shape of the mitochondrial network in cells; and (iii) mitochondrial quality control through regulation of autophagy and mitophagy [40]. In liver homogenates, by Western blot analysis, no significant difference in the ratio pAMPK/AMPK was detected in the liver of BTBR mice compared to controls (Figure 6E), while a significant reduction in pACC/ACC ratio and CPT1 expression levels was observed (Figure 6F).

### 3.5. Hepatic Oxidative Stress and Antioxidant/Detoxifying Defense in BTBR Mice

**Figure 7:**
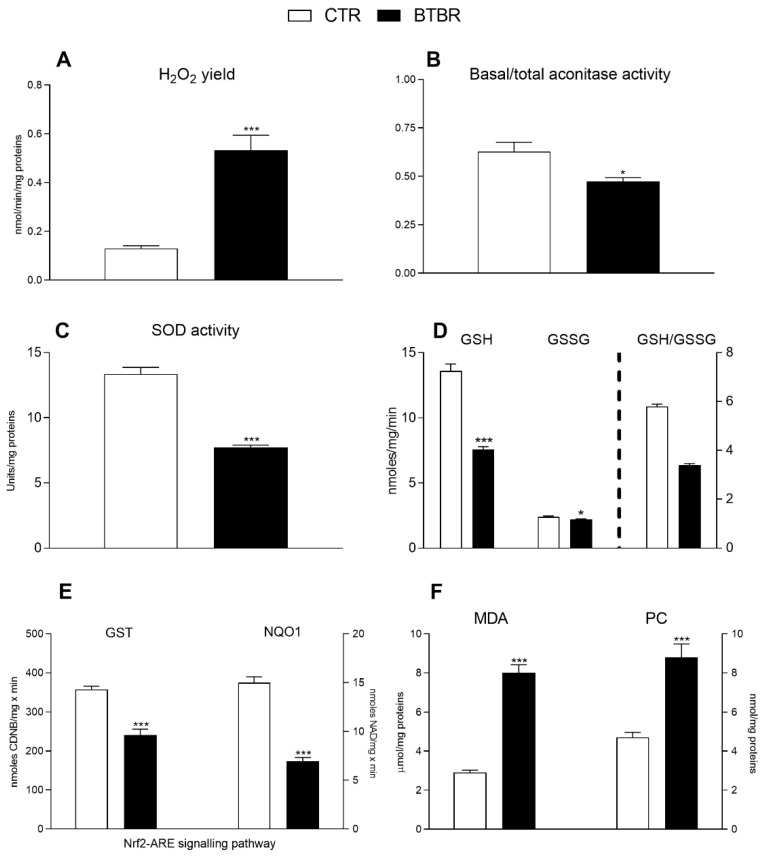
Hepatic oxidative stress profile and antioxidant/detoxifying defenses in control and BTBR mice. Hydrogen peroxide (H_2_O_2_) release (**A**), basal/total aconitase (**B**) and superoxide dismutase (SOD) activity (**C**) were determined in hepatic isolated mitochondria. Reduced glutathione (GSH), glutathione oxidized (GSSG) content ((**D**), left Y axis) and ratio GSH/GSSG ((**D**), right Y axis), glutathione transferase (GST) and NAD(P)H Quinone Dehydrogenase (NQO1) levels (**E**) were evaluated in hepatic tissue. Hepatic levels of malondialdehyde (MDA) and protein carbonyls (PC) (**F**). Data are presented as means ± SEM from *n* = 7 animals/group. * *p* < 0.05, *** *p* < 0.001, BTBR vs. Control (CTR).

Compared to the controls, BTBR mice showed an increase in oxidative stress indicated by their significantly higher H_2_O_2_ production in isolated mitochondria (Figure 7A) and their lower mitochondrial enzymatic activities of both aconitase (a sensitive marker of oxidative stress) and SOD (the first line of defense against oxidative stress) (Figure 7B,C). The liver redox status was determined by measuring the hepatic GSH and GSSG concentrations. The results showed that both GSH and GSSG content significantly decreased in the BTBR animals compared to controls (Figure 7D, left Y axis). Consistently, the BTBR mice exhibited a lower GSH/GSSG ratio compared to controls (Figure 7D, right Y axis). In addition, the detoxifying activities of the enzymes GST and NQO1 were significantly lower in BTBR animals compared with the other group (Figure 7E). BTBR mice, compared to controls, exhibited a higher content of hepatic MDA and protein carbonyls (Figure 7F), indicating their greater degree of hepatic lipid peroxidation and protein oxidation.

## 4. Discussion

Our results provide compelling evidence that the altered metabolic profile of BTBR mice, characterized by inflammatory status and oxidative stress, is linked to hepatic alteration due at least in part to mitochondrial dysfunction. 

ASD etiopathology involves environmental factors triggering metabolic alterations in genetically sensitive individuals. One of these abnormalities is the brain mitochondrial dysfunction, which is observed in a significant subset of ASD patients [18,19] and in an animal model [22]. Furthermore, it was demonstrated that the alterations of brain mitochondrial activity and the consequent oxidative stress are intimately associated with the activation of the immune inflammatory pathways, which are linked to the severity of symptoms [41]. The mitochondria are involved in the control of cellular bioenergetics, but they also represent the primary driver for the metabolic processes and the maintenance of energy and inflammatory balance for the body. These organelles can produce pro- or anti-inflammatory signals, influencing the levels of ROS [42].

In this work, we demonstrated the involvement of hepatic mitochondrial dysfunction in BTBR mice as an ASD animal model, suggesting mitochondria as target for innovative strategies aimed at counteracting metabolic and inflammatory alterations characterizing ASD. Our data showed an increase in inflammatory state, body weight, lipid gain, dyslipidemia and a higher food intake in BTBR mice compared to their controls, clearly denoting an alteration of metabolic profile in this animal model. The increase in the ratio body weight gain/energy intake indicates a more efficient energy utilization in BTBR animals vs. controls, which can be partially responsible for the increase in body lipid. With body weight gain and accumulation of fat mass, leptin level increases, while adiponectin decreases [43]. Leptin, a critical regulator of food intake and energy expenditure, is an inflammatory adipokine whose dysregulation has been proposed as a mechanism involved in brain pathologies. Indeed, elevated levels of circulating leptin are consistently found in neurodevelopmental disorders including ASD [44]. Adiponectin, like leptin, is heavily involved in the control of energy homeostasis and inflammation [45]. We observed decreased adiponectin levels in BTBR mice, which is linked to inflammatory state as shown by increased levels of TNF-α, IL-1β, IL-6, LPS and MCP-1 in the serum and liver of these animals. Notably, among the inflammatory factors, serum TNF-α levels are doubled in the BTBR. Interestingly, this cytokine inhibits adiponectin secretion and is tightly correlated with inflammation and oxidative stress [46]. These increased serum levels of inflammatory cytokines in BTBR mice are in agreement with previous results in ASD patients [6], and they are linked to the worsening of ASD symptoms, suggesting a relationship between peripheral immune activity and altered behaviors in ASD [47]. BTBR mice also display altered microbiota composition, which is one of the features of ASD [13]. As known, the gut microbiota has coevolved with the host influencing harvest, storage, and energy expenditure [17]. The microbiota composition is modified in obese humans and mice, and it rapidly changes in response to dietary factors, increasing the capability to extract energy from the diet [48]. Although it is not possible yet to define a gut microbial signature for ASD, several evidence demonstrated a decreased Bacteroidetes/Firmicutes ratio in ASD patients [49], similarly to those reported in a diet-induced obese animal model [50]. This evidence suggests that the dysbiosis in BTBR mice may increase their capacity to extract the energy from the diet, which can partially contribute to the increase in the metabolic energy efficiency of these animals. Dysbiosis in BTBR mice can trigger a persistent gut inflammation that contributes to systemic inflammation, releasing harmful mediators that reach the liver through the portal vein [17]. Consistently, our data demonstrated increased serum levels of LPS and MCP-1 and hepatic inflammation (i.e., increased transaminases, TNF-α and IL-6 hepatic levels). The hepatic inflammation was confirmed by morphological analyses showing in BTBR mice liver damage characterized by steatosis, an increase in Kupffer cells, and numerous ultrastructural alterations (i.e., crenulate nucleus, nuclear inclusions, hypertrophic nucleoli, and congested rough ER). Nuclear inclusions are a common finding in hepatocytes with liver diseases, including non-alcoholic fatty liver disease [51,52], but they are also associated with the aging process [53]. We hypothesized that congested RER in the liver of BTBR mice is linked to ER stress. Indeed, we detected in the liver of BTBR animals an increased expression of BIP and p-EIF2α. Accordingly, it has been previously demonstrated that the proliferation of the RER is correlated to cellular stress in patients affected by liver diseases [54]. Intercommunication between the ER and mitochondria can be involved in the modulation of both energy metabolism and ER stress [55]. Indeed, these alterations are associated with several pathological conditions, including obesity and metabolic syndrome [56]. Intriguingly, the ER–mitochondria interface is involved in the regulation of mitochondrial fission and inflammasome formation [57]. Accordingly, in various inflammatory diseases, inflammation leads to an increase in mitochondrial fission (small and fragmented mitochondrial morphology), mediated by DRP1 and FIS1 proteins [58]. In line with the study of Ahn et al. [27] on cortical brain tissues from BTBR mice, no significant difference was observed in the DRP1 expression levels among the two groups. However, by electron microscopy, we observed an increased number of small-sized mitochondria in the liver of BTBR mice, suggesting mitochondrial fission, which was also confirmed by the higher expression levels of FIS1 in the BTBR group compared to controls. In addition, typically, mitochondrial fission is associated with dysfunctions of these organelles, which are characterized by increased ROS production [59]. Thus, our results, showing a shift toward fission in the hepatic mitochondria of BTBR animals, is in line with the decreased mitochondrial function and increased fragmentation reported in ASD patients [19]. Moreover, the presence of numerous mitochondria associated with ER, observed by electron microscopy, can be related to the increased expression levels of MFN2 in the liver of BTBR animals. This protein is involved in mitochondrial dynamics, but it is also able to directly mediate ER–mitochondria tethering in mammals [60]. This increased levels of MFN2 is in agreement with previous study demonstrating a higher expression of the MFN2 gene in the oral mucosa in children with ASD [61]. However, a different pattern of the MFN2 expression levels was shown in the brain of ASD patients [62,63]. Altogether, these results suggest that MFN2 expression is tissue-dependent [60].

The increased liver inflammatory state may be the cause or the consequence of altered hepatic mitochondrial functions [9]. We observed that BTBR mice exhibited a reduced respiratory capacity compared to control animals. State 3 respiration significantly decreased both in the presence of FADH-linked (succinate) and in FADH and NADH-linked substrates (malate and palmitoyl carnitine). A decrease in state 3 of the mitochondrial respiration may be due to defects in the activity of substrate oxidation reactions (complex II, complex III, complex IV, and dicarboxylate carrier) and/or in the activity of the phosphorylation reactions (ANT, ATP synthase and phosphate carrier). In the presence of succinate plus FCCP, we observed a decreased oxygen consumption in the liver of BTBR mice, suggesting that the impairment of mitochondrial functions is not related to phosphorylation reactions but depends on altered substrate oxidation [64]. Furthermore, the damaged hepatic mitochondrial functions in BTBR animals are confirmed by the reduced β-oxidation capacity in the presence of malate and palmitoyl carnitine substrates. This decrease could be attributable, at least in part, to the decreased phosphorylation of ACC, leading to high levels of malonyl-coA, which negatively regulates fatty acid β-oxidation by inhibiting CPT1, which catalyzes the transport of long-chain fatty acyl-CoAs into the mitochondria. The reduced activation of ACC in BTBR mice does not parallel with the significant activation of AMPK, suggesting that different pathways are involved. Interestingly, in primary dermal fibroblasts from ASD patients, an overactive mitochondrial bioenergetics was reported. The authors speculated that these changes could represent adaptive mechanisms to sustain an increased energetic demand, perhaps resulting from a chronic oxinflammatory status [65].

In our study, BTBR mice showed an increased degree of coupling and subsequently an increased mitochondrial efficiency. Mitochondria generate ATP through the oxidation of the nutrients, and the energy generated by the electron transport is utilized to phosphorylate ADP to ATP. The electron transport and the ATP synthesis are processes that are closely coupled, but some of the energy generated by electron transport is uncoupled from ATP synthesis. Part of the proton gradient across the inner membrane is dissipated without generating ATP [66]. Instead, a high degree of coupling found in BTBR mice induces an increased mitochondrial energy efficiency. This increase in energy efficiency implies that fewer substrates need to be burned to obtain the same quantity of ATP. As a consequence, the excess of unburned substrates promotes lipid deposition. The partial block of electron flow within the respiratory chain, due to the impairment of the respiratory chain (demonstrated by the decrease in state 3), and the decrease in mitochondrial uncoupling both contribute to the increase in oxidative stress (increased MDA, PC and aconitase activity) in BTBR mice. The uncoupling is a major mechanism for the adjustment of the membrane potential to control mitochondrial ROS emission. With a mild uncoupling, the mitochondria can avoid the excessive supply of electrons/reducing equivalents in the respiratory complexes and minimize the probability of electron interaction with oxygen [66]. Furthermore, the BTBR animals also showed a decrease in the activity of SOD enzyme, which is the first line of defense from oxidative stress [67]. Concomitantly with the decrease in the enzymatic antioxidant activities in these animals, we also observed a decrease in detoxifying activities (GST and NQO1) in liver, with a consequent worsening of the cellular redox state (GSH/GSSG).

Our data show that the hepatic steatosis in ASD mice depends, at least in part, on mitochondrial dysfunctions and an increase in mitochondrial coupling, which is associated with an increase in body weight gain/energy intake ratio. Indeed, the more efficient energy utilization in BTBR animals contributed to the decreased mitochondrial oxidation of energy substrates, especially fatty acids, with a deleterious consequence on intracellular triglycerides accumulation and lipotoxicity. These alterations could explain, at least in part, the increased incidence of metabolic syndrome in ASD patients. In addition, these metabolic dysfunctions may exacerbate the inflammatory profile and worsen the ASD symptoms.

## 5. Conclusions

We observed, in BTBR mice, an altered metabolic profile concomitant to altered serum levels of hormonal and inflammatory markers. In these mice, we reported a liver damage characterized by steatosis, an increase in Kupffer cells, and numerous ultrastructural alterations including congested and stressed endoplasmic reticulum. The increased liver inflammatory state is related to the altered hepatic mitochondrial function and the shift toward fission in mitochondrial dynamics. The mitochondrial impairments contribute to the oxidative stress exacerbated by a decrease in the enzymatic antioxidant/detoxifying activities in these animals, with a consequent worsening of the cellular redox state. Altogether, our results suggest that hepatic mitochondrial dysfunction and oxidative stress exacerbate meta-inflammation in autism. This conclusion may help with understanding the pathophysiology of ASD and contribute to identifying hepatic mitochondria as targets for the development of innovative treatments of ASD.

## Data Availability

Data is contained within the article.
